# Embryonic Onset of Sexually Dimorphic Heart Rates in the Viviparous Fish, *Gambusia holbrooki*

**DOI:** 10.3390/biomedicines9020165

**Published:** 2021-02-08

**Authors:** Seyed Ehsan Mousavi, G. John Purser, Jawahar G. Patil

**Affiliations:** 1 Fisheries and Aquaculture Centre, Institute for Marine and Antarctic Studies, University of Tasmania, Taroona, TAS 7053, Australia; john.purser@utas.edu.au; 2 Inland Fisheries Service, New Norfolk, TAS 7140, Australia

**Keywords:** sexual dimorphism, somatic organ, heart rate, ventricle size, embryo, adult, viviparous, *Gambusia holbrooki*

## Abstract

In fish, little is known about sex-specific differences in physiology and performance of the heart and whether these differences manifest during development. Here for the first time, the sex-specific heart rates during embryogenesis of *Gambusia holbrooki*, from the onset of the heart rates (HRs) to just prior to parturition, was investigated using light cardiogram. The genetic sex of the embryos was post-verified using a sex-specific genetic marker. Results reveal that heart rates and resting time significantly increase (*p* < 0.05) with progressive embryonic development. Furthermore, both ventricular and atrial frequencies of female embryos were significantly higher (*p* < 0.05) than those of their male sibs at the corresponding developmental stages and remained so at all later developmental stages (*p* < 0.05). In concurrence, the heart rate and ventricular size of the adult females were also significantly (*p* < 0.05) higher and larger respectively than those of males. Collectively, the results suggest that the cardiac sex-dimorphism manifests as early as late-organogenesis and persists through adulthood in this species. These findings suggest that the cardiac measurements can be employed to non-invasively sex the developing embryos, well in advance of when their phenotypic sex is discernible. In addition, *G. holbrooki* could serve as a better model to study comparative vertebrate cardiovascular development as well as to investigate anthropogenic and climatic impacts on heart physiology of this species, that may be sex influenced.

## 1. Introduction

Sexual dimorphism is a common feature in most vertebrates and is more readily recognised with external phenotypes and/or behaviour [1]. This dimorphism also extends to physiology and functions of internal somatic organs, which are not directly involved in reproductive processes [2].

Teleosts display a spectacular array of sexual dimorphism [3]. Specifically, like most Poeciliids, *Gambusia holbrooki* adults exhibit striking sexual dimorphism [4]. For instance, adult *G. holbrooki* display body size differences with males smaller than females, and the anal fin of males is modified into a gonopodium that acts as an intromittent organ [5]. However, sexual dimorphism of internal organs other than the gonads is poorly understood in this species. There is increasing evidence suggesting that such dimorphism in the heart of juvenile [6] and adult [7,8] zebrafish, *Danio rerio*, liver of adult medaka, *Oryzias latipes* [1], and swim-bladder of adult South Asian torrent minnows (*Psilorhynchus*) [9], exists. These studies are also complemented by recent molecular studies where gene/s exhibit sexual differences at the transcription level in multiple tissues other than gonads [10], including the spleen of adult *G. holbrooki* [11]. Transcriptomic sex-dimorphism in the liver of adult *O. latipes* has also highlighted sex-specific differences in response to several physiological processes [1]. Moreover, cardiac regeneration and immune-related responses, post-injury of zebrafish hearts [12], are among the most sexually dimorphic. Sex differences in physiology are commonly attributed to developmental and/or hormonal factors, but there is evidence that cell-intrinsic mechanisms also play important and persistent roles [13]. These biological sex differences in gonadal and non-gonadal tissues manifest downstream of the inherent chromosomal determinants [2,14]. Nonetheless, how early and when during development these sex-dimorphisms manifest and shape the sexual identity of adults remains far more obscure.

The heart is the first definitive organ to develop and become functional during embryogenesis, as any later survival depends on its proper function [15,16]. Like in other teleosts, the heart of Poeciliids has two chambers, i.e., single-chambered atrium and ventricle [17], but both cellular and molecular mechanisms of heart contraction are similar to mammals [16,18]. Part of a complex reproductive adaptation in Poeciliids is a placenta-like specialised tissue suggesting a closer evolutionary link to placentation in mammals [19]. Therefore, studies on heart and physiology in Poeciliids could answer longstanding questions on sex differentiation, evolution, and developmental mechanisms, as well as assisting in better understanding human cardiac diseases [20]. Of the Poeciliids, *G. holbrooki* and *G. affinis* are the best examples of adaptation and survival under extreme environmental conditions [21] and are currently considered highly invasive species worldwide [22]. Such adaptive ability demands functional plasticity in organ development and their performance, with the heart the most critical for survival [23]. Studies in even the less hardy Poeciliids such as guppy, *Poecilia reticulata*, and Amazon molly, *Poecilia formosa*, have shown that they are less prone to heart diseases [17,24]. More importantly, in the guppy and Amazon molly, degenerative changes in the heart muscle (i.e., myocardium), and a marked loss of muscle fibres in the bulbous arteriosus, are known to resemble symptoms observed in ageing mammals [24]. Therefore, Poeciliids, in general, are of biomedical interest [25], with *G. holbrooki* and its sister species, *G. affinis*, attracting additional evolutionary and ecological interest as a result of their wide distribution, short life cycle, and invasiveness [22].

In higher vertebrates, sex-specific differences in cardiac performance and physiology are well documented [14,26]. Although cardiac studies have been conducted increasingly in zebrafish [16,18] and medaka [27,28,29], no study to date has examined sex-specific heart rates (HRs) in fish embryos. For instance, the influence of sex on ventricular function and size in juvenile and adult zebrafish [7,8,30], and HR in adult sockeye salmon, *Oncorhynchus nerka* [31] and rainbow trout, *Oncorhynchus mykiss* [32], have been reported. Interestingly, turtle embryos exposed to thyroid hormones [33] and temperature regimes [34] are known to demonstrate a sexually dimorphic cardiovascular physiology that manifests before hatching, with no comparable studies in fish.

There is also increasing evidence suggesting that several aspects of cardiac and gonadal development occur in parallel. For example, in embryonic zebrafish migration of primordial germ cells (PGCs) towards the putative gonadal ridge coincides with heart formation by 24 h post-fertilisation [35]. Moreover, a recent study indicated that there is a relation between primitive testis development with that of heart and liver in embryonic zebrafish [23], suggesting that many aspects of organogenesis are closely related and those obviously may thus bear witness to more cryptic sex differentiation events that occur in parallel.

Based on preliminary observations of two distinct clusters of HRs in developing embryos of the same developmental stage and that of sexually dimorphic ventricle size in adults of this species, it was hypothesised the cardiac dimorphism may occur during early development and could be linked with the sex of the individuals. To test this hypothesis HRs of *G. holbrooki* through ontogeny to adulthood was investigated.

## 2. Materials and Methods

### 2.1. Fish Collection, Maintenance, and Sample Preparation

**Figure 1:**
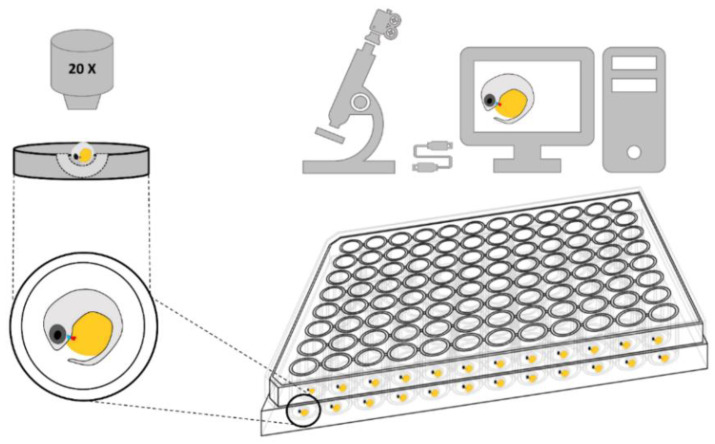
Illustration of embryos in 96-well microplate for stereomicroscope imaging and video recording. Embryos were allocated individually into each well and immobilised in pre-prepared ‘U’ shaped depression in agarose block with the heart facing towards the eyepiece.

Mature females with gravid spots were collected from the Tamar Island Wetlands Reserve (41°23.1′ S; 147°4.4′ E), Launceston, Tasmania and transported to a molecular laboratory in Taroona, Institute for Marine and Antarctic Studies, University of Tasmania. Fish were maintained in recirculating aquaria (NO_2_^−^/NO_3_^−^ ≤ 0.1 ppm, NH_4_^+^/NH_3_^−^ ≤ 0.5 ppm, temperature: 25 ± 1 °C; salinity: 0 ppt; 16L:8D photoperiod) acclimatised for at least one week prior to use. Sexually mature fish were fed twice daily to satiation with commercial fish pellets (TetraMin^®^ tropical micro granules, Melle, Germany) and freshly hatched Artemia nauplii. Feeding was suspended four hours prior to experiments. One hundred females based on the size and a range of gravid spot intensity values [22] were selected, individually euthanized (100 ppm AQUI-S^®^), dissected under stereomicroscope and embryos collected and staged [36]. Embryos at early organogenesis (EO), mid-organogenesis (MO), late organogenesis (LO), early pharyngula (EP), late pharyngula (LP), and just prior to parturition (JPP) were used to conduct the HR measurements (n = 10, 20, 50 or 100 embryos per stage were used depending on the assay). Either a top (early, mid, and late organogenesis) or lateral view (later developmental stages—early and late pharyngula and just prior to parturition stages) was used for observations. At those viewing planes, the heart is visible clearly and did not require the application of pigment inhibitors and anaesthesia. Live embryos were washed with 1X PBS (Phosphate-buffered saline) and dispensed individually into precast ‘U’ shaped agarose wells in 96-well plates (Figure 1). Then, each well was filled with 50 µL filtered (0.2 µm) and sterilised water from recirculating aquaria.

### 2.2. Heart Rate Determination by Direct Visual Count

Concurrent to video recording and direct visual counts (VC) of heartbeats were made manually for 1 min under the microscope. For greater accuracy, a secondary manual verification of heartbeats was undertaken by counting the beats on slow-motion videos generated in Premiere Pro CC 2020 (14.0.3, Adobe™ software, San Jose, CA, USA).

### 2.3. Acquisition of Heartbeats for Digital Motion Analysis

#### 2.3.1. Video Acquisition System

The HR and its frequencies were determined using a non-invasive method previously described for adult zebrafish [7] with modifications (Figure 1). Briefly, a 96-well, flat-bottom, microplate (Greiner Bio-One North America Inc., Monroe, NC, USA) platform with a light scattering panel to cast a stable field of illumination throughout the wells was employed. Room temperature was maintained at 25 ± 0.5 °C and cold light source was used for illumination. For observation, the embryos were always orientated with anterior-to-left and were immobilised in ‘U’ shaped agar wells to minimise pixel intensity (PI) fluctuations (Figure 1). As each well was moved into position, light was applied from a halogen source for 70 s. The first 10 s of illumination served to acclimate the embryos to the bright light conditions. Following acclimation, videos of stationary embryos were captured at sampling frequency of 60 frames per second (fps), with 2592 × 1944-pixel resolution for the 60 s duration at each developmental stage (EO–JPP). An automated Leica dissecting microscope (MZ12.5, Leica Microsystems, Wetzlar, Germany) equipped with a remotely controlled camera (Dino-Eye Edge series AM7025X, Dino-Lite Digital Microscope, New Taipei, Taiwan) recorded videos at 20× magnification (Figure 1).

#### 2.3.2. Video Processing

**Figure 2:**
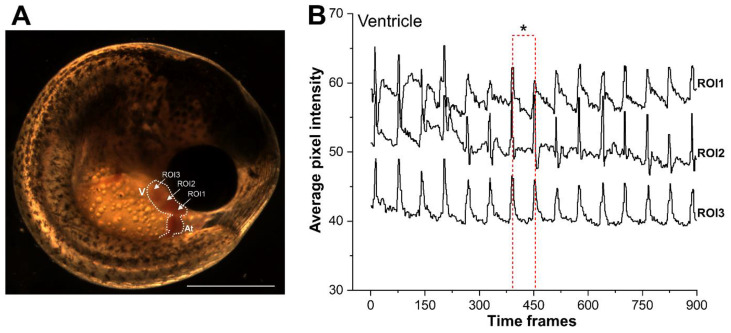
Representative ventricular ROIs with their corresponding light cardiograms (LCGs). The heart (white dotted line) of a developing embryo at (**A**) pharyngula (lateral view) with atrium (At) and ventricle (V) marked. (**B**) Representative graph illustrating the LCGs at three different regions of interest (ROIs) (1–3) within the ventricle. The time interval (marked *) between two consecutive ventricular peaks remained consistent between selected ROIs. The peak and trough in each tracer correspond to ventricular systolic and diastolic phase, i.e., emptying and filling of blood cells, respectively. ROI = region of interest; scale bar = 1 mm.

All captured videos were exported into Adobe Premiere Pro CC software for processing. The video recording frame rate and resolution were reduced from 60 fps to 30 fps and 2592 × 1944 pixels to 640 × 480 pixels, respectively. Edited videos were then individually imported to ImageJ (version 1.52u, National Institutes of Health, Bethesda, MD, USA). The region of interest (ROI) between ventricle and atrium were chosen to check the signal quality and beating ratio. Multiple ROIs (e.g., ROI1, ROI2, and ROI3) were chosen in both ventricle and atrium (Figure 2A) for cross verification. At least ten ROIs were selected for each ventricle and atrium by using a circle tool. A time series analyser V3 plugin v. 3.0. (ImageJ, version 1.52u, National Institutes of Health, Bethesda, MD, USA) was used directly to analyse the light cardiogram (LCG) changes in the selected ROIs. The average pixel intensity changes correspond to ventricle and atrium contractions over time (s) defined as light cardiogram (LCG).

#### 2.3.3. Beat Interval, Beats Per Minute (BPM), and Heart Rate Frequency (Hz)

A two-step approach was developed that derived HRs of embryos based on the changes in LCG resulting from contraction and relaxation of the heart during each heartbeat (Figure 2B). The first step involved building LCG profiles using semi-automated image analysis of videographs generated for each embryo. Given that heart tissue is more opaque than the static background, contraction of the heart increased average brightness within the prescribed ROI; conversely, relaxation decreased average brightness (Figure 2). The algorithm recorded these regular oscillations in brightness as LCG. In the second step, the LCG vs. time data were converted to a frequency spectrum using the Fast Fourier Transform (FFT) algorithm, a method applied previously to determine HR in fish embryos [37] and adults [7]. HR corresponds to heart frequency (HF).

The signal quality for ROIs within both ventricle (Figure 2A,B) and atrium were cross verified. As the average pixel intensity can be affected by factors such as red blood cells, pigments, and debris, ROIs were selected in the heart region with minimal noise and maximum stability (Figure 2B). To exclude pixel intensity oscillations caused by non-heart tissue, a minimum threshold value of 50 was applied, then the data were smoothed using the Savitzky–Golay function in OriginPro 2020 (Originlab Corporation, Northampton, MA, USA). This threshold value was identified after a sensitivity analysis was performed over a subset (n = 20/stage) of acquired videos (by the construction of a cumulative histogram of pixel intensities for ventricle and atrium). The average signal intensity at chosen ROIs between ventricle and atrium were compared and analysed using a peak analyser and Gauss model. Briefly, the LCG and time series data were normalised using inbuilt options (0–100) and (0–1), respectively. Both ventricle and atrium data were smoothed using an FFT algorithm at a cut-off frequency of 0.2 Hz. To determine the number of peaks and bases, the Quick Peak (Gauss model) and peak analyser functions were used. The peak and base finder option was set to detect both positive and negative tracers with the second derivative method (i.e., to search for hidden peaks and bases). The time duration (s) between ventricular systolic (contraction) and atrial diastolic (dilation) phase constituted a complete cardiac cycle. The time delay between the atrioventricular (A-V) peak values of the extracted synchronous chronologies within the same cardiac cycles were measured using time history values for ventricular and atrial peaks. The A-V delay time, defined as the time duration (s) between the onset of the atrial peak and the onset of the subsequent ventricular peak, represents the resting phase of the heart. The average resting time per minute for each developmental stage was calculated by multiplying the total A-V delay/sec by 60.

The two-chambered embryonic (i.e., pharyngula stage) heart and typical light-cardiogram (LCG) with corresponding ventricular ROIs (1–3) are presented in Figure 2. The beat interval was calculated by subtracting the average time between two consecutive peaks, and BPM was obtained by dividing one minute (60 s) by the time interval [7]. The frequency spectra were obtained by applying an FFT algorithm in OriginPro 2020 (Originlab Corporation, Northampton, MA, USA) to the time histories (n = 50/stage), from which the cardiac rhythm of each embryo was identified, i.e., rhythms that correspond to the F_dominant_ (F_D_) of the spectrum. 

### 2.4. Validation of Digital Heartbeat Counts

The digital cardiac counts were validated against the respective visual counts for all developmental stages (n = 100/stage). Linear correlation/regression analyses of mean HR (bpm) obtained from automated LCGs and those determined by direct visual counts were undertaken. For subsequent comparative analysis (between sex and stages), the BPM data from only ventricles were used, as ventricles were relatively easier to locate owing to their larger size. Moreover, there was no significant difference between the contraction rates of ventricle and atrium.

### 2.5. Genetic Sexing of Gambusia Embryos at Different Stages

#### 2.5.1. DNA Extraction and PCR Condition

Post-HR determination, the HR data were segregated into two groups (high and low HRs) using the heat map algorithm (OriginPro 2020, Originlab Corporation, Northampton, MA, USA). Then, a subset of embryos (n = 10/sex/developmental stage) were randomly chosen and subjected to genetic verification of sex. Briefly, a small piece of the caudal fin was clipped from each embryo using a microscalpel, post euthanasia. Genomic DNA was extracted using the MyTaq™ Extract-PCR Kit (BIO-21126, Meridian Life Science, Inc., Cincinnati, OH, USA) according to the manufacturer’s instructions. The genetic sex of the individuals was determined by polymerase chain reaction (PCR) using male and female-specific genetic markers as described [11,38]. Briefly, PCR mix (10 μL) comprised of 1 × MyTaq™ HS Red mix (Meridian Life Science, Inc., Cincinnati, OH, USA), 1.0 μM of each primer and 50 ng of genomic DNA template. Thermal cycling (T100™ Thermal Cycler, Bio-Rad Laboratories, Inc., Gladesville, NSW, Australia) consisted of 95 °C for 1 min, followed by 30 cycles of 95 °C for 5 s, 60 °C for 5 s, and 72 °C for 20 s. Female and male specific amplicons were separated using gel electrophoresis.

### 2.6. HR and F_D_ Determination in Sexually Mature Fish

**Figure 3:**
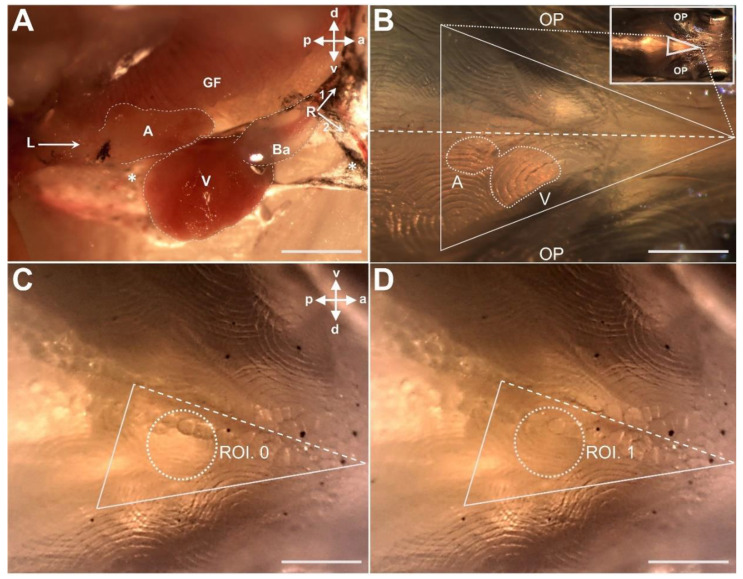
Photographs showing the location and orientation of heart in a dissected (**A**) and live (**B**–**D**) adult female, *Gambusia* *holbrooki*. (**A**) The directions of blood flow into (left arrow) atrium (**A**) and out (right arrows) through bulbous arteriosus (Ba) are shown. The ventricle (V) and epicardium (asterisks) are also marked. Posterior to left and anterior to the right. (**B**) Ventral views of an anaesthetised fish secured in place under a stereomicroscope. The location of the heart and both chambers are shown. (**C**,**D**) Photographs were directly extracted as time-sequence image stacks from video corresponding to consecutive ventricular diastolic (ROI. 0, brighter) and systolic (ROI. 1, darker) states. The isosceles juxtaposes triangular space between the opercula that can serve as a marker to locate the beating heart in vivo, i.e., to the left of the ventral midline (dashed line) of the fish. (**C**,**D**) are magnified views of (**B**) in the respective two consecutive image stacks, where the right-angled triangles correspond to the lower half of the isosceles (not to proportion). a, v, p, and d represent anterior, ventral, posterior, and dorsal, respectively. OP = operculum. GF = gill filaments. (**A**,**B**), scale bar = 1 mm. (**C**,**D**), scale bar = 500 μm.

Video recording of heartbeats was done according to the method recently described for adult zebrafish [7]. Briefly, ten sexually distinct female (147.5 ± 30.3 mg, 23 ± 2.8 mm) and ten male (140 ± 47.5 mg, 23 ± 2.1 mm) fish were chosen based on their phenotype, i.e., the presence of gonopodium in males and gravid spot in females. Following anaesthesia (2.5 ppm AQUI-S^®^ for 7–10 min), the fish was positioned ventral side up with gills and opercula immersed in water (Figure 3B). At that viewing orientation, heartbeats were visible through the scales and skin with the ventricle closer to the ventral surface (Figure 3B). Fish were first acclimatised to the lighting conditions (30 s) and videos were captured for 1 min at 60 frames/s (fps) at 25 ± 0.5 °C. Room temperature was maintained at 25 ± 0.5 °C using a controlled heater and cold light source was used for illumination. Video acquisition was completed within 5 min after inducing anaesthesia. Following the video recording, the location of the heart (i.e., ventricle) was identified, and ROIs were chosen (Figure 3C,D). An isosceles triangle was placed between the opercula, with the ventral midline of the body perpendicular to the base of the triangle connected to its vertex (Figure 3B–D) and LCG data acquired. The isosceles triangle and ventral midline served as a guide to locate the heart and thus ROI assignment. Following LCG, fish were placed in an aerated recovery chamber containing fresh water from the system without anaesthetic and monitored. Fish generally recovered within ∼3 to 5 min, and there were no mortalities. After recovery, the sex of each fish was genetically confirmed using male and female-specific genetic markers (described in the Section 2.5.1).

### 2.7. Size Comparison of Adult Male and Female Explanted Hearts

Sexually mature *G. holbrooki* (n = 25/sex) were selected based on secondary sexual characteristics as above. Adults were individually euthanized (100 ppm AQUI-S^®^), standard length (mm) and weight (mg) were measured using an ocular micrometre under a dissecting microscope and an SE2 ultra-microbalance (Sartorius AG, Göttingen, Germany), respectively. The hearts were dissected (Figure 3A) using microscalpel.

**Figure 4:**
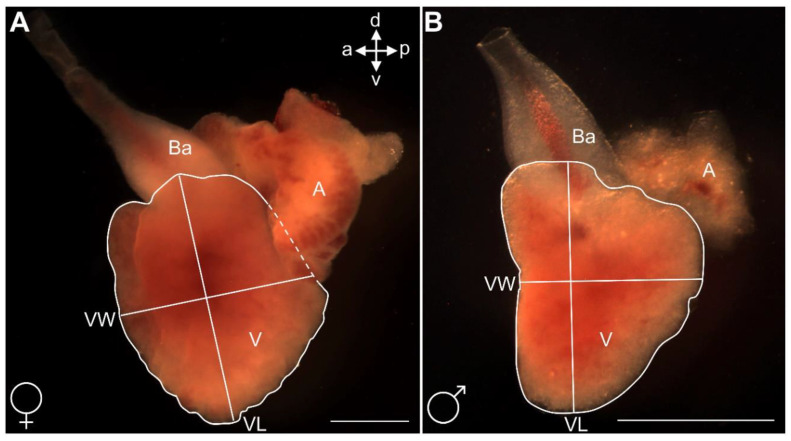
Freshly explanted hearts of adult (**A**) female and (**B**) male *G. holbrooki*, with schematic measurements of ventricle length (VL) and width (VW). The solid lasso line outlines the ventricle boundaries laterally. Bulbous arteriosus (Ba), ventricle (V), atrium (A). The horizontal (VW) and vertical (VL) lines correspond to the width and length of the ventricle. a, v, p, and d represent anterior, ventral, posterior, and dorsal, respectively. Scale bars = 500 μm.

Fish and hearts were photographed using a stereomicroscope (Leica MZ12.5, Leica Microsystems, Wetzlar, Germany) and camera (Leica DFC420, Leica Microsystems, Wetzlar, Germany) with its associated software (LAS version 3.8.0, Leica Microsystems, Heerbrugg, Switzerland). Photographs of hearts were analysed using ImageJ. The ventricle length (VL) was measured from its apex to its junction with the bulbous arteriosus (Figure 4A,B). The ventricle width (VW), was then measured by drawing a perpendicular line (Figure 4A,B) at the midpoint of the VL extending to the edges of the ventricle [39]. To quantify ventricle size, both ventricle surface area (mm^2^) and volume of the ventricle (mm^3^) were calculated as per the protocol previously described [40] using ImageJ. Ventricle volume measurements were normalised by condition factor (a) derived separately for each sex (male = 0.01; female = 0.02) using the allometric growth equation, W = aL^3^. Prior to analysis, ventricle and eviscerated body mass data were log_10_ transformed.

### 2.8. Statistical Analysis

All plots were generated, and statistical analysis carried out in OriginPro 2020 (Originlab Corporation, Northampton, MA, USA). Data are presented as means ± SD. A two-way ANOVA was used to compare means within and between developmental stages and sexes, with post-hoc Tukey’s multiple comparisons test assessing pair-wise differences. Where applicable, a student *t*-test was also applied to assess differences between paired-means. The Gauss model was used to analyse the normal distribution of peaks and bases of both ventricle and atrium separately. Significant difference generated by the Gauss model was tested by the chi-squared test (χ^2^). Linear regression analysis (r^2^ and slope) and Pearson’s correlation analysis (R-value) determined goodness of fit between visual and automated LCG HR counts. Difference between two means was considered significant when *p* < 0.05–0.001 (* *p* < 0.05, ** *p* < 0.01, *** *p* < 0.001).

## 3. Results

### 3.1. Comparison and Validation of Heart Rates: Automated vs. Manual Assessment

**Figure 5:**
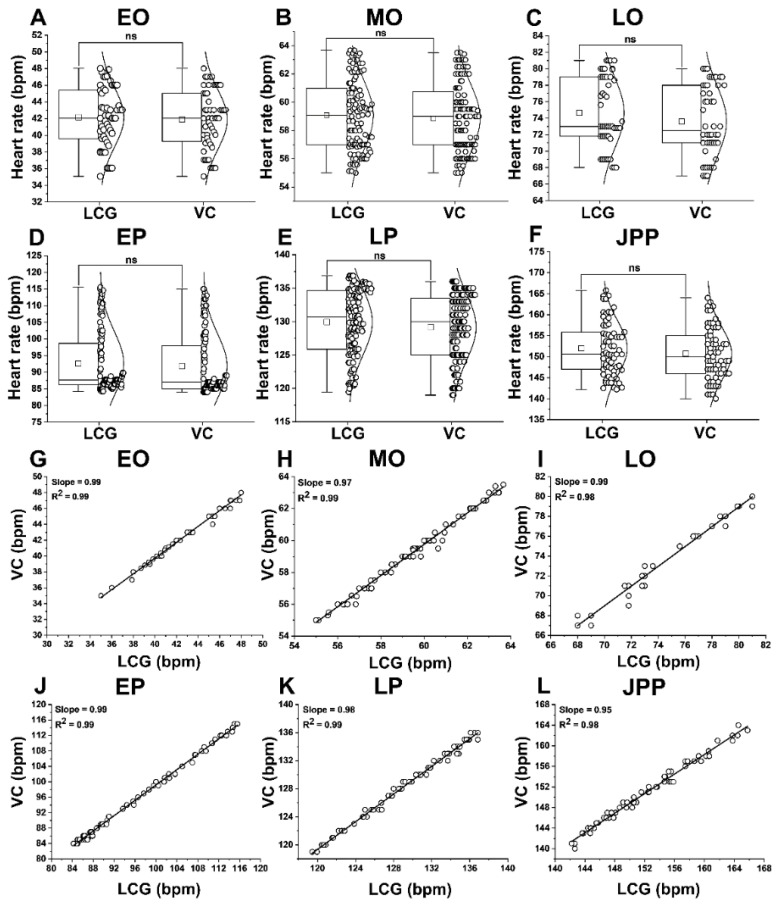
Box plot (**A**–**F**) and linear relationship (**G**–**L**) between visual and LCG counts of heart rate (HR) at six different developmental stages of *G. holbrooki*. There was a significant correlation (*p* < 0.05) between the two HR counts, at the respective developmental stages. VC = visual count; LCG = light cardiogram analysis; ns = non-significant. Box plot data are presented as means ± SD. The horizontal line in each box plot separates the data distribution into upper (50%) and lower (50%) quartiles. Small square and a horizontal line on each box plot indicate mean and median values, respectively (n = 100 embryos per each developmental stage).

The HR in wild-type *G. holbrooki* embryos at different stages (EO, MO, LO, EP, LP, and JPP) using ventricular LCG (42.7 ± 7.3, 59.5 ± 3.5, 75.5 ± 6, 86.7 ± 5.8, 128 ± 7, and 153 ± 12 bpm) and visual counts (41 ± 6, 58 ± 4, 74 ± 6, 78 ± 7, 127 ± 8, and 152 ± 11 bpm) were comparable (*p* > 0.05) for the respective stages (Figure 5A–F). Similarly, strong linear correlation (*p* < 0.05) between the HR determined by visual counts and LCG was found, and the slope of linear regression was nearly 1 (0.95–0.99) in all stages (Figure 5G–L).

### 3.2. Light Cardiograms of Ventricle and Atrium Mutually Validate

**Figure 6:**
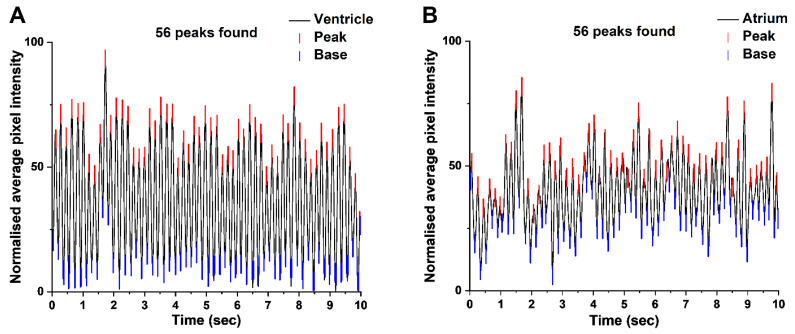
Representative LCGs of the ventricle (**A**) and atrium (**B**) of embryonic (just prior to parturition (JPP) stage) *G. holbrooki* heart. Graphs represent smoothed and normalised average PI. Quick Peak (*Gauss*) and peak analyser functions were used to identify the number of peaks. Red and blue bars mark the peak and base, respectively. In this instance, the numbers of peaks for both chambers were the same (56) and there were no significant differences at this stage and among others tested (*p* ≥ 0.05, n = 20 for each developmental stage).

Based on LCGs, the cardiac rhythm of the atrium and ventricle of the embryos at the same developmental stage were not significantly different (*p* ≥ 0.05) from each other, i.e., the number of peaks per second were comparable (Figure 6). It was easier to obtain ventricular data owing to its relatively larger size and higher normalised pixel intensities (i.e., amplitude) of LCG tracers (Figure 6A) compared to those of the atria (Figure 6B). Therefore, subsequent comparative analyses between developmental stages relied on ventricular data.

### 3.3. Synchronicity in Ventricular and Atrial Contraction

**Figure 7:**
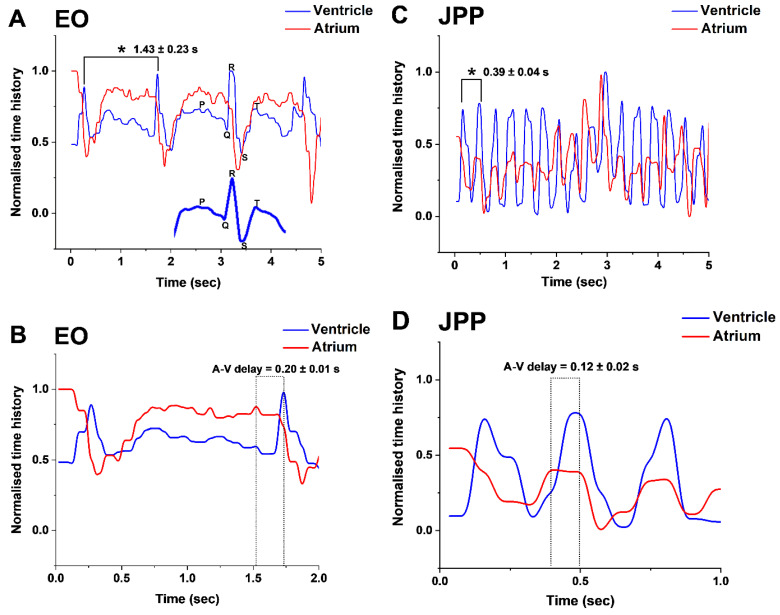
Light-cardiograms (LCGs) representing synchronicity of contractions and relaxations of cardiac compartments at early organogenesis (EO) and JPP of *G. holbrooki*. The chronograms (**A**,**C**) represent normalised ventricle and atrium time history contractions over 5 s at EO and JPP stages, respectively. Smoothed partial LCG corresponding to synchronous ventricular and atrial contractions resembling a typical ECG (P-wave, QRS-complex, and T-wave) chronograms is presented (A inset). The complete cardiac cycle, i.e., the time interval between two consecutive ventricular peaks, is marked with an asterisk. (**B**,**D**) are magnified view of (**A**,**C**) respectively showing time delay (dashed boxes) between atrium and ventricle beats (i.e., A-V delay).

As expected, the quantitative analysis of synchronous time histories of the atrium and ventricle yielded two chronologies (Figure 7); one each for atrium and ventricle. The resulting LCG profile (Figure 7A) resembled that of a typical ECG with characteristic P-wave, QRS- complex and T-wave (Figure 7A inset). The comparison of time history between two consecutive ventricular peaks (i.e., one cardiac cycle), for all the embryonic stages, reveals the time (s) taken to complete a cardiac cycle decreased significantly with advancing stages (*p* < 0.05, n = 10 for each stage; Figure 7). This ranged from a high of 1.43 ± 0.23 s in EO to 0.39 ± 0.04 s in JPP (Figure 7A,C, respectively). Similarly, the atrium-ventricle (A-V) delay decreased from 0.20 ± 0.01 s in EO to 0.12 ± 0.02 s in JPP (Figure 7B,D, respectively). Although the average delay between atrial and ventricular beats (A-V delay) reduced with advancing embryonic stages, the total average A-V delay (s) per minute increased (*p* < 0.05, n = 10 for each stage). The total average A-V delay (s) over 5 s of the LCGs was 0.60 ± 0.02 and 1.8 ± 0.3 s at EO and JPP stages respectively (Figure 7), with the cardiac resting time three-fold higher in the latter (7.2 ± 0.1 and 21.6 ± 0.3 s/min for EO and JPP stages, respectively).

### 3.4. FFT Transformed Heart Rate Frequency (F_D_) Validate HR Counts and Their Increase with Advancing Developmental Stages

**Figure 8:**
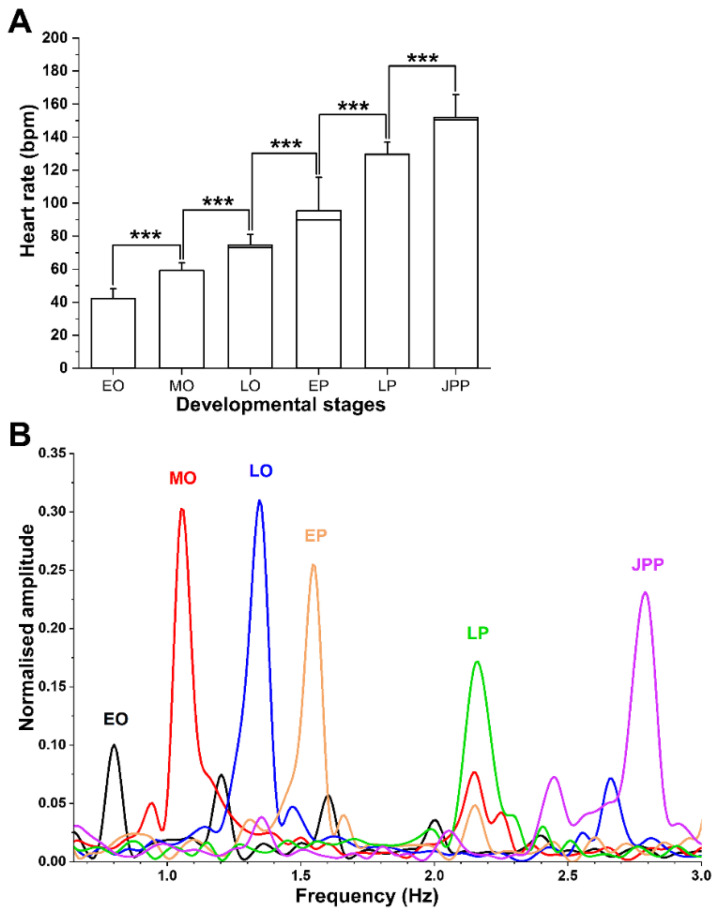
Graphs illustrating increasing heart rates and frequencies in advancing developmental stages of *G. holbrooki*. (**A**) The heart rate of embryos (n = 50/stage) at each developmental stage (EO, mid-organogenesis (MO), late organogenesis (LO), early pharyngula (EP), late pharyngula (LP), and JPP) showed significantly higher HR compared to the preceding stage. The average beats (mean ± SD) per minute (bpm) is shown (*** *p* < 0.001). (**B**) The average FFT transformed dominant heart frequency (F_D_) for the six different embryonic stages also increased with advancing developmental stages and were consistent with the HR counts.

The HR ranged from 42 at EO to 153 bpm at JPP stages (Figure 8A). A significant increase in HR with advancing development stage was found (*p* < 0.05). With the lowest HR of 42.7 ± 7.3 bpm at EO, a progressive and significant increase to 59.5 ± 3.5, 75.5 ± 6, 86.7 ± 4.8, 128 ± 8, 153 ± 15 bpm at MO, LO, EP, LP, and JPP stages, respectively, was observed (Figure 8A). FFT transformed time intervals into frequency domains are presented in Figure 8B.

The F_D_ of each embryonic stage were consistent (*p* ≥ 0.05); however, these also increased (*p* < 0.05) with advancing developmental stages. The shift in F_D_ (Figure 8A) were consistent with those of HR (Figure 8B).

### 3.5. Cardiac Rate Was Influenced by the Genetic Sex of the Embryos

**Figure 9:**
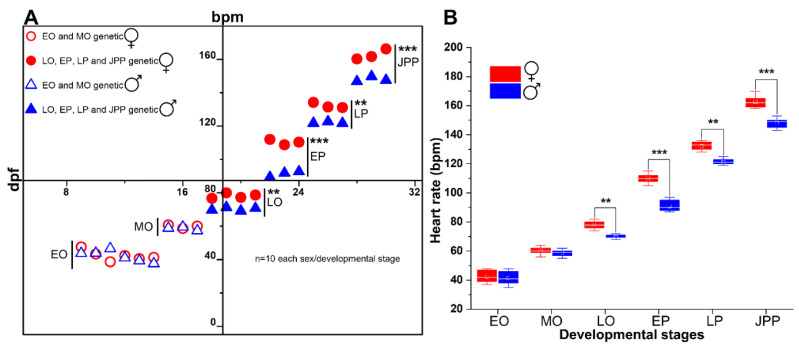
Graphs showing sex-specific differences in average heart rate with advancing embryonic stages of *G. holbrooki*. Both scatter (**A**) and box plots (**B**) show significant differences between HRs of males and females (n = 10/sex), except for EO and MO. (**A**) shows three different groupings of data, reflecting age (days post fertilisation; dpf), HR (bpm), and genetic sex of embryos. The onset of sex-specific differences in HR was at LO stage. All data are expressed as means ± SD. Values with asterisks are significant (** *p* < 0.01, *** *p* < 0.001). Small square and a horizontal line on each box plot indicate mean and median values, respectively.

When the data for each developmental stage was portioned based on genetic sex, a clear difference in average HR between the two sexes was evident, with females having higher HRs (** *p* < 0.01, *** *p* < 0.001) at all developmental stages except at the two earliest stages (EO and MO) examined (Figure 9). The male and female HRs at EO and MO clustered together, while at LO a clear separation in the male and female grouping could be observed (Figure 9A). This separation was most conspicuous at EP (*p* < 0.001) and remained so in the following stages, although at later stages (i.e., LP and JPP) this separation was less conspicuous but significant (*p* < 0.05). This trend of sex and age-specific differences in HRs was also evident from the box plot (Figure 9B). The average HR (bpm) for males and females were not significant at EO and MO (*p* ≥ 0.05). However, from LO onwards, the HRs were significantly different between males and females (*p* < 0.01 and *p* < 0.001, n = 10 each sex at each embryonic stage). The sex-specific difference was the highest at EP and JPP (*p* < 0.001) compared to LP (*p* < 0.01). The average A-V delay time was not significantly different between males and females at respective developmental stages (*p* ≥ 0.05, n = 10 each sex at each embryonic stage).

### 3.6. Sex-Biased Cardiac Frequency in Developing Embryos

**Figure 10:**
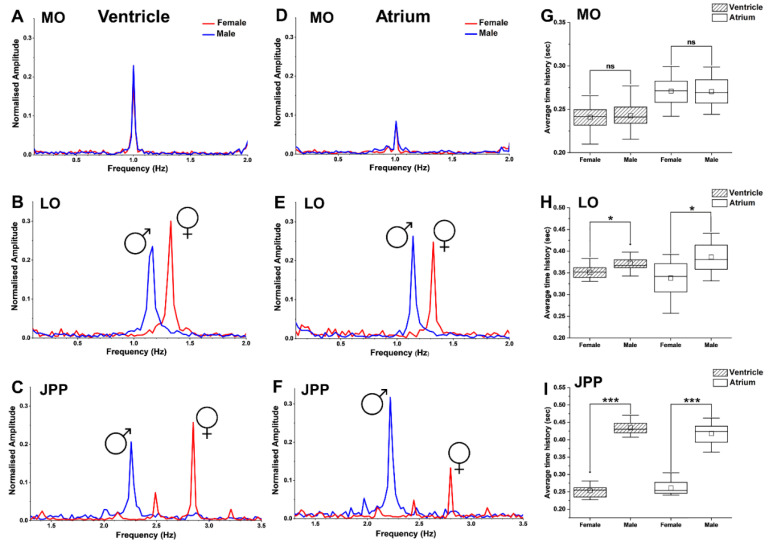
Graphs showing cardiac frequency (F_D_) analysis of genetic male and female *G. holbrooki* embryos at three developmental stages. Plots (**A**–**C**) represent ventricular F_D_ of the female/male at MO, LO, and JPP stages, while (**D**–**F**) represent those of atrium at the corresponding stages, respectively. Box plots (**G**–**I**) illustrate the average time history (s) differences between ventricle and atrium contractions in male and female embryos at MO, LO, and JPP stages, respectively. Box plot data are presented as means ± SD (n = 10 /developmental stage/sex). Small square and a horizontal line on each box plot indicate mean and median values, respectively. ns = non-significant. * *p* < 0.05 and *** *p* < 0.001.

The F_D_ between male and female embryos were also significantly different (*p* < 0.05) starting from LO. This was consistent for both ventricle and atrium frequencies (Figure 10; only MO, LO, and JPP stage data is presented). At MO, the atrium and ventricle frequencies (F_D_) of both males and females were identical (Figure 10A,D). However, from LO onwards these frequencies increased (*p* < 0.05) in a sex-specific manner, i.e., significantly higher in females than males. The onset of sex-biased F_D_ differentiation was first observed at the LO stage (Figure 10B,E) and became more pronounced at JPP (Figure 10C,F) which correspond to the HR differences (Figure 9B) between male and female embryos at these stages. In agreement with this observation, the average time history (s) between two consecutive ventricle or atrium contractions in males was significantly higher than female embryos at both LO (*p* < 0.05, Figure 10H) and JPP (*p* < 0.001, Figure 10I) stages (n = 10 at each stage). However, these were not significantly different at MO (*p* ≥ 0.05, n = 10, Figure 10G). Indices of systolic and diastolic functions were not significantly (*p ≥* 0.05, n = 10) different over time between embryos in both sexes at MO (Figure 10A,D). However, the onset of cardiac systolic and diastolic differentiation between sexes was observed in embryos at LO stage (*p* < 0.05, Figure 10B,E) and this trend became more pronounced in atrium diastolic function in male embryos (*p* < 0.05, Figure 10F) and ventricle systolic function in female embryos at JPP (*p* < 0.05, Figure 10C).

### 3.7. Sexual Dimorphism in HR and F_D_ of Adults

**Figure 11:**
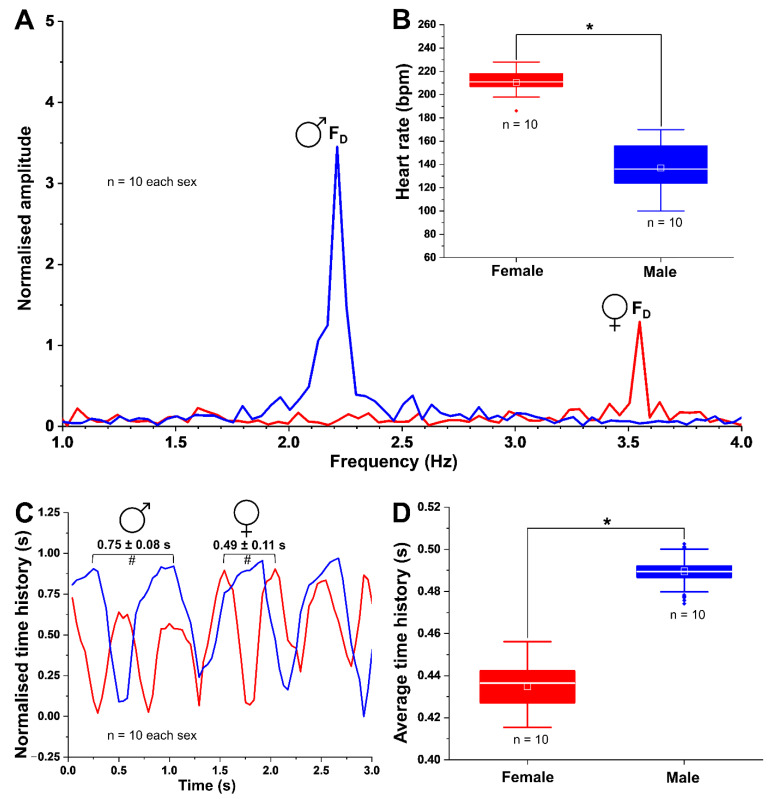
Graphs representing the sex-specific differences in average F_D_ (**A**), HR ((**B**) inset), cardiac cycle period (**C**), and time history between ventricular contraction (**D**) in adult males and females of *G. holbrooki*. (**C**) the cardiac cycle period, i.e., the time interval between two consecutive ventricular peaks, is marked with # for both sexes. Box plot data are presented as means ± SD (n = 10/sex). Values with an asterisk are significant (* *p* < 0.05). Small square and a horizontal line on each box plot are shown mean and median values, respectively.

A clear difference in average HR of the two sexes in adults was evident with females having higher HRs (*p* < 0.05) (Figure 11A,B). The average heart beats per min (bpm) for female and male fish were 204.4 ± 14.69 and 141.4 ± 26.66, respectively. As expected, cardiac F_D_ in females was also higher (*p* < 0.05, n = 10 each sex) compared to males (Figure 11A). Conversely, the cardiac amplitude was higher in males than females (Figure 11A). The normalised amplitudes between individuals of the same sex were not significantly different (*p* ≥ 0.05, n = 10 each sex).

A complete cardiac cycle length was 0.49 ± 0.11 s and 0.75 ± 0.08 s for sexually mature females and males, respectively (Figure 11C). This cardiac cycle was significantly higher (*p* < 0.05, n = 10 each sex) in male than female fish (Figure 11C). Furthermore, peak analysis showed that the number of ventricular peaks within 3 s of cardiac contractions in adult female was ∼2 times higher (*p* < 0.05, n = 10 each sex) that of the male fish. Consistent with this observation, the average time history (s) between two consecutive ventricle contractions in males was significantly higher than females (Figure 11D; *p* < 0.05, n = 10 each sex). Interestingly, F_D_ in adult male fish (∼2.2 Hz) remained unchanged from those observed in male embryos at JPP stage (Figure 10C,F). Nevertheless, the F_D_ of the female embryos at JPP (∼2.8 Hz) was approximately 75% that of the adult females (∼3.5 Hz). In addition, significant (*p* < 0.05) differences in ventricular amplitude (i.e., diastolic state) was evident (Figure 11A) between females (1.29 ± 0.58) and males (3.45 ± 0.87). The results (Figure 11C,D) also show that the heart (i.e., ventricle) of males (0.75 ± 0.08 s) required more time for contractions and relaxations than females (0.49 ± 0.11 s). As determined by FFT data, there were significant differences in HF and HR between adult male and female individuals.

### 3.8. Morphology and Size of Adult Male and Female Heart

**Figure 12:**
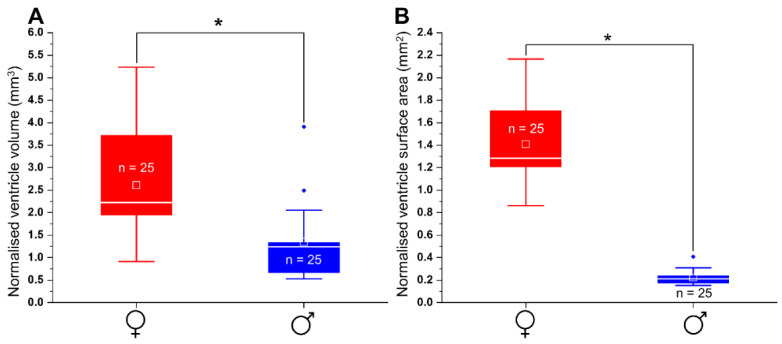
Box plots showing (**A**) normalised volume (mm^3^) and (**B**) surface area (mm^2^) of the ventricle in adult female and male of *G. holbrooki*. The ventricle volume (mm^3^) and surface area (mm^2^) were significantly higher in adult female compared to male (*p* < 0.05, n = 25/sex). Small square and a horizontal line on each box plot indicate mean and median values, respectively. All data expressed as the means ± SD. * *p* < 0.05.

The observation of external cardiac morphology in adult male and female fish shows that the entire heart is contained within the epicardium, which exhibits sparse pigmentation, whereas the heart chambers exhibit no pigmentation (Figure 3). The ventricle appears conical, more so when viewed laterally. The atrium is horseshoe-shaped and envelopes the ventricle, covering the dorsal region (Figure 4A,B). The conical shape of the ventricle and horseshoe shape of the atrium were more pronounced in female than male individuals (Figure 4). The pear-shaped bulbous arteriosus was located dorsally and anterior to the ventricle and points superior rostrally towards the gills while the outflow tract tapers to become the ventral aorta (Figure 3A). Consistent with the sexual size dimorphism in the species, the size of the normalised (to growth condition factor) heart (i.e., ventricle) sizes of females were significantly larger than those of the males (n = 25/sex, *p* < 0.05, Figure 12).

## 4. Discussion

Previous research has demonstrated that LGC can be used to measure HR and F_D_ of embryonic zebrafish [41]. More recently, the technique was also demonstrated to be applicable for adult zebrafish [7], which we show here to be applicable to all life stages of *G. holbrooki*. Importantly, the study also suggests that the cardiac sex-dimorphism manifests as early as late-organogenesis and persists through adulthood in *G. holbrooki*. This appears to be the first study to demonstrate the early onset of cardiac sex-dimorphism in any teleost species.

### 4.1. The LCG Mimics ECG Morphology and Enables Reliable Determination of the Heart Rate (HR) and Frequency (HF) in Embryonic and Sexually Mature G. holbrooki

As observed, the heart of embryonic *G. holbrooki*, exhibits the rhythmic patterns of contraction and relaxation, in a fashion similar to those reported in zebrafish, *D. rerio* [16] and medaka, *O. latipes* [28], with blood flows from the sinus venosus into an atrium then pumping through to the ventricle and out via the aorta (Figure 3A).

The resemblance of *G. holbrooki* LCG morphology to the electrocardiogram (ECG) of the human heart, with all the principal and cyclical components—P-wave, QRS-complex, and T-wave [42]—with rapid activation of the ventricle signal confirms shared mechanisms and thus the utility of LCGs as a non-invasive tool. Importantly, this was applicable to both adults and embryos. Typically, in ECG, P-wave represents the syncytial contraction of the atrial muscle followed by a considerably larger QRS-complex corresponding to that of the thicker ventricular muscle [43], with the interval between the P- and T-waves representative of the A-V delay, i.e., the time taken for the action potential to traverse between the atrium and ventricle [44]. The resemblance of LCG profiles in *G. holbrooki* to those of ECG in zebrafish [45] and humans [46], suggests these can be used to interpret depolarisations and repolarisations states of the heart during the cardiac cycle reliably, as has also been verified in zebrafish [45]. Moreover, the LCG can differentiate between atrial and ventricular signals which is otherwise overwhelmed by stronger ventricular signal (contraction) in case of ECG [44]. In agreement with the previous studies in embryos [47] and adult [7] zebrafish, an empirically high correlation occurred between the LCG and VC heart rate in *G. holbrooki*, further strengthening the reliability of the LCG technique. There are also technical advantages to use LCG as this can be recorded with relative ease and automated for recording and analyses [7].

Except for zebrafish [16,18] and medaka [27,28], there has not been any comprehensive cardiac study in other teleosts, particularly Poeciliid species, until now. The heartbeat and blood flow of *G. holbrooki* embryos could be readily visualised, with stable and regular HR for six hours post-removal from the ovarian sac, allowing robust and repeat recordings. Importantly, the range of embryonic HRs of *G. holbrooki* (42–153 bpm) were much closer to those of normal fetal (110 to 150 bpm) [48] and adult (60–100 bpm) [49] humans. In contrast, the HRs of the commonly used model species such as zebrafish (120–180 bpm) and mice (300–600 bpm) [37] are relatively much higher.

Reasons for comparatively closer HRs of *G. holbrooki* to those of humans are yet unknown. However, it is possible that the shared internal fertilisation with a placenta-like reproductive strategy [19] may in part explain the evolutionary conservation of the heart function [50] of *G. holbrooki* and placental vertebrates. Although the teleost lineage exhibits morphological evolutionary novelty such as bulbus arteriosus, they express genes that are conserved across vertebrates [50]. Therefore, fish such as zebrafish [18] and medaka [28,29] have been used as the model for cardiovascular studies. However, these species do not appear to show cardiac-related sex-specific differences, unlike humans [14,26]. Based on our observations, *G. holbrooki* may serve as a superior animal model to investigate sex-specific cardiovascular differences that may be relevant to human conditions. Typically, the relatively large size of the litter (clutch) would facilitate better replication of studies. Moreover, livebearers produce more robust offspring that also minimise sample to sample variations [22] as was observed in this study.

### 4.2. Cardiac Rate Increases with the Progression of Embryonic Development

Early in organogenesis, a cardiomyocyte contraction is first observed when the primitive heart tube is being formed around 9 dpf in *Gambusia* sp. [36], as was also observed in this study. The heart of *G. holbrooki* in early development, exhibits a slow contraction. Perhaps, at this time, the myocardial cells of the primitive tube are automatic (i.e., spontaneously depolarise), slowly conduct the electrical impulse, and unlike zebrafish, may have developed sarcomeres and sarcoplasmic reticulum, leading to strong contraction properties. Nevertheless, the observed cardiac contractions in *G. holbooki* indicate a more co-ordinated pattern even when the heart is still a primitive linear tube (i.e., early-organogenesis), unlike zebrafish embryos [16], where the onset of cardiomyocyte contractions are irregular and uncoordinated at early stages of heart development. However, as *G. holbrooki* embryos develop, at later stages, the heart contractions show a more co-ordinated pattern as is also the case for zebrafish embryos [16], which is linked to a substantial increase in cardiomyocyte numbers [51]. This is also consistent with observations in medaka [27,29] and mammals including humans [52].

It is remarkable that the LCG profile is also markedly similar to that of an ECG. The distinct LCG diastolic and systolic signatures of *G. holbrooki* (Figure 7), resembling those of domestic chicken, *Gallus gallus* [53], and zebrafish [15,45], suggests that the heart is a valve-like structure, which prevents retrograde flow during development, as also described for zebrafish [18,51].

Unlike zebrafish [15,16], the occurrence of an early mature heartbeat in *G. holbrooki* is comparable to those of chicken and mice embryos [46] whose pacemaker activity is initiated even before the first heartbeat, when the mature components of the cardiac conduction system (CCS) are not morphologically recognisable, as yet. Moreover, CCS shows remarkable evolutionary conservation among taxa [54]. In the human foetus, the slow contractions and relaxations characteristic of the myocytes in the atrioventricular contractions (AVC) prevent blood from flowing back into the atria during ventricular activation and contraction, a role later adopted by the mature atrioventricular (A-V) valves [46]. Similarly, in *G. holbrooki*, the slow contractions in early heart development may indicate a shared mechanism to ensure unidirectional blood flow. Subsequent increases in contraction rates were consistent of changes from a peristaltic to sequential contractions of the atrium and ventricle as is also known to occur in zebrafish [16,18]. However, in *G. holbrooki*, ventricular and atrial contractions were distinguishable at very early stages of heart formation, similar to those of chicken and mice [46]. Nevertheless, the decrease of cardiac cycle length whilst shortening of cardiac time intervals (diastole and systole) with advancing embryonic development is consistent with that of zebrafish [15].

The accentuation of the LCG profile at the tail-end (T-wave) is likely due to atrial systolic augmentation of ventricular filling, mimicking those reported for other vertebrates including humans [15,16]. In parallel, the consistently higher and rhythmic ventricular systolic peaks compared to those of the atrium is indicative of a pressure gradient caused by numerous resistance points. For example, differences in atrioventricular systolic peaks are associated with the pressure gradient caused by the resistance to flow across the developing cushions between the ventricle and the bulbus arteriosus [55]. Besides, the decline in the early diastolic state, during a cardiac cycle, to near zero in JPP compared to EO, implies that wall stress changes considerably at the later developmental stage, suggesting a progressive change in ventricular wall thickness. The establishment of distinct diastolic tracers at JPP resembling those of adults, suggests that a valve-like structure has already formed between the ventricle and atrium, as was also inferred in zebrafish [15,16].

The LCG profiles of early developmental stages with a characteristically wide QRS-complex trace correspond well with studies in the African lungfish, *Protopterus annectens* [56], and crocodiles, *Alligator mississippiensis* [54], where the atrial conduction system is as yet undeveloped at that point. The occurrence of such primitive cardiac tracers has been attributed to the functional presence of Purkinje fiber network during avian [57] and mammalian [58] embryonic development, a pattern not readily obvious in zebrafish [59].

The atrioventricular (A-V) delay of *G. holbooki* is consistent with the time required to eject the atrial blood into the ventricle before its contraction. Typically, this role is performed by the A-V node which delays electrical impulses originating in the atria before they are allowed to excite the ventricle, thereby allowing coordinated contraction of the atria before ventricular systole and facilitating forward blood flow [60]. This A-V delay coincides with the transition from a peristaltic contraction of the heart tube into a more efficient sequential contraction of the atrium followed by the ventricle [16]. In zebrafish, cardiomyocytes are positioned circumferentially around the A-V canal [61], suggesting that morphological differences between cardiomyocytes of the A-V canal and the myocardial chamber contribute to the A-V delay. Moreover, a subsequent increase of the A-V delay (e.g., from ∼0.1 to 0.2 s in EO to JPP, respectively, in this study) with corresponding HR increases allows adequate diastolic filling and in turn leads to an improvement in cardiac ejection [16,18].

The significant increase in A-V delay with advancing embryogenesis corresponds well with cardiac chamber development in zebrafish [61], where the A-V myocardium first differentiate into slow conducting tissue [62] and then differentiate to become precursors of the A-V node at the ventricular apex [62]. This then establishes a fully mature and faster conducting myocardial network transmitting an apex-to-base activation pattern [18]. Such specialised cardiac conduction produces intracardiac hemodynamic forces that can influence cardiac development and also plays a vital role in cardiac chamber morphogenesis [18] to assure efficient compartmentalisation of oxygenated and deoxygenated blood within the ventricle [63]. A lack of a morphologically distinct chamber structure during early development does not necessarily imply that the A-V delay function is absent. For example, in chick embryos, morphologically distinct bundles are not present before stage 31, i.e., 7 days of incubation, but the precursors of these structures can be identified much earlier as a diffused network [64]. The A-V delay in *G. holbrooki* is remarkably similar to the embryonic chick heart [65], suggesting an A-V delay node (i.e., conduction network) could be already formed in *G. holbrooki* even before the chambers become morphologically distinct.

The significant increase in the HR (bpm) with their corresponding F_D_ across embryonic development in *G. holbrooki* is in agreement with those previously reported for zebrafish [16,37] and medaka [28]. These shared patterns of increasing heartbeat for normal embryonic development are necessary to meet the rapid growth and expansion of the circulatory system of their growing embryos. For instance, heart contraction increases as embryonic development progress to ensure the perfusion of all tissues of the growing embryo [37]. The formation of a functional vertebrate heart requires processes that progressively fashion correct morphology and size during embryonic development [16,18]. In particular, adequate size and wall thickness of the heart chambers are necessary for generating enough blood circulation for growth of the embryo [16]. Increasing HR and arterial blood pressure during embryonic development has previously been documented in other vertebrates, e.g., the desert tortoise, *Gopherus agassizii* [66], the American alligator, *A. mississippiensis* [67] and the emu, *Dromiceius novaehollandiae* [68]. Likewise, in normal human foetuses between 6 and 13 weeks of gestation, the HR increases with gestational age—gradually, from 87 to 189 bpm at 38 to 62 days of gestation, respectively, suggesting that an increase in HR (bpm) during ontogeny is a fundamental requirement for maintaining convective transport in embryos that are increasing in body mass [69].

### 4.3. Cardiac Rate of G. holbrooki is Influenced by the Sex of the Embryos and Adults

Our findings suggest that the sex dimorphism in HR (bpm) of *G. holbrooki* embryos is unique or may yet support the prevailing lay view that in human foetuses the HRs of the female foetuses are considerably faster than male at the second and third gestational periods [70]. The sex-specific differences in HR of *G. holbrooki,* are also in part similar to those reported for chicken [71] and snapping turtles, *Chelydra serpentina* [34], where female embryos exhibited higher mean HRs than males. However, these differences in *G. holbrooki* were distinct with no overlap in the HR ranges of female and male embryos allowing the inference of sex of the individuals as early as late-organogenesis. At this developmental stage, the gonads are known to be sexually differentiated in Poeciliidae [72] including *G. holbrooki* (personal communication, Ngoc Tran and Komeil Razmi) as well as other freshwater teleosts [1], suggesting a close association between sex and HRs in many, if not all fish species. Similarly, in *C. serpentina*, the sex-dimorphic HR and arterial blood pressure coincide with the chronology of gonad differentiation, steroidogenesis, and associated metabolic rates, including growth [34].

Phenotypic sex differences in live-bearing fish such as guppy [73] as well as mammals arise from the unequal effects of sex chromosomes [74]. Such effects can occur either directly, due to differential gene expression from the X and Y chromosomes in XX and XY of the non-gonadal cells; or indirectly, via the gonads, resulting in hormonal differences throughout life [14]. In the last 35 years, evidence has accumulated suggesting that some sex differences in non-gonadal tissues (e.g., cardiac) are caused by the differential effects of X and Y genes within the respective tissues [2,14,74]. Sexual dimorphism of internal organs other than the gonads is rare, but it is known to occur in invertebrates such as *Drosophila melanogaster* [13], and vertebrates including some species of fish [1,8]. Several studies show regulation of sexual dimorphism in aggression [75], growth, and size-at-maturity [76] by Y-linked genes in some Poeciliid species. It is therefore possible that the sex-linked genes may have a direct role in phenotypic traits of the *G. holbrooki* heart, as is also known to occur in mice and humans [2,74].

The occurrence of higher HRs and diastolic function in female *G. holbrooki* coincides with a corresponding larger size of the heart (ventricle). Sexually dimorphic differences in cardiac size have also been reported in adult zebrafish [8,30] although with differences only in diastolic function, i.e., contractility. The differences in heart function of adult female and male in *G. holbrooki* could also be attributed to structural differences other than heart size. For example, differences in ventricular wall thickness can cause sex-specific differences in the heart function in humans [77].

Ventricular filling (i.e., end-diastolic volume) is determined by various factors, including atrial contraction, end-systolic volume, the pressure gradient, wall compliance which sets the amount of pressure needed to stretch the wall, and HR which sets the time available for filling [44]. Ventricular and atrial inner volume may be sex-specific in *G. holbrooki* that manifest early during development and persist throughout adulthood. In *G. holbrooki*, although ventricular size in adult males is smaller than females, the ventricle requires more time to fill with lower diastolic contractility. Thus, it could be possible that males rely more on passive cardiac filling (i.e., ventricular elastic recoil during relaxation to suck blood into the chamber), whereas females may rely more on active filling, presumably aided by the thicker and stiffer ventricular wall as well as larger volume. This explains the higher beat rates in females to compensate for their specific structural and physiological need.

Functionally, these smaller chamber compliance of adult males may be cardioprotective, by allowing the cardiac wall to counteract the increased haemodynamic stress encountered during high cardiac load [78]. Regardless, the sex-specific diastolic cardiac outputs (males lower than females) in adults is in agreement with the ventricular size differences in adult zebrafish [8,30], and wall thickness [79] and changes in cardiomyocyte contractile properties [80] in humans.

*G. holbrooki* females with higher HR could benefit from a faster rate of repair post-cardiac injury, as higher HRs facilitate the cardiomyocyte proliferation under normal and regenerating conditions [81]. Consistent with this, female hearts regenerate more rapidly than male hearts [12], as is also known to occur following estradiol treatment with a concurrent increase of HR [82] in zebrafish embryos. Besides, the higher HR in female *G. holbrooki* may be necessary to improve the blood flow to their relatively larger body size (than male) as well as to nourish individual embryos during gestation. Conceivably, however, the higher proliferation of cardiomyocytes as the result of higher HR in females could elicit faster cardiac ageing and make them more prone to diseases than males. For instance, female guppies are more prone to lesions of the bulbus arteriosus [17], an aspect that might apply to all Poeciliid species including *G. holbrooki*.

## 5. Conclusions

This study demonstrated age- and sex-dependent differences in heart rates and diastolic function of *G. holbrooki*. Importantly, the study also suggests that the cardiac sex-dimorphism manifests as early as late-organogenesis and persists through adulthood. This appears to be the first study to demonstrate the early onset of cardiac sex-dimorphism in any teleost species. The sex difference in heart rate is useful to predict the embryonic sex and for its automation using optical sorting and machine vision system. An ability to rapidly sex developing embryos is particularly important for monitoring the sex ratio where mono-sex production is desirable. We also suggest that these findings highlight the utility of *G. holbrooki* as a model for comparative cardiovascular studies in vertebrates as well as to investigate anthropogenic and climatic impacts on heart physiology of this species, that may be sex influenced.

## Data Availability

Not applicable.
